# Self and parent-reported sleep difficulties of adolescents with childhood conduct problems and comorbid psychological problems

**DOI:** 10.1192/j.eurpsy.2021.609

**Published:** 2021-08-13

**Authors:** M. Tomasiello, A. Martin-Storey, V. Bégin, M. Déry, M. Poirier, C. Temcheff

**Affiliations:** 1 Educational And Counselling Psychology, McGill University, Montreal, Canada; 2 Psychoéducation, Université de Sherbrooke, Longueuil, Canada; 3 School Of Criminology, Université de Montréal, Montreal, Canada; 4 Secteur Disciplinaire Des Sciences De L’éducation, Université du Québec à Rimouski, Rimouski, Canada

**Keywords:** Conduct problems, sleep, comorbidity, longitudinal

## Abstract

**Introduction:**

Children with conduct problems (CP) exhibit problematic externalizing behaviors that violate the rights of others and/or societal norms, are likely to present with comorbid psychological problems, engage in high-risk behaviours during adolescence and in turn, display poorer prospective health in adulthood. However, little known about their adolescent quotidian behaviors, such as their sleep behaviours, which may contribute to these poorer outcomes.

**Objectives:**

Using a sample designed to assess the longitudinal consequences of CP, the current study examines how histories of CP and comorbidity with depressive symptoms and/or attention-hyperactivity problems are associated with sleep difficulties during adolescence.

**Methods:**

744 participants from an ongoing longitudinal study in Québec, Canada were assessed for CP and comorbidities when they were 6 to 10-years-old. They were classified as without CP, CP only, CP and depressive symptoms, CP and attention-hyperactivity problems, or CP, depressive symptoms and attention-hyperactivity problems based on parent and teacher-reported indices. Sleep difficulties were assessed 7 years later, using self and parent-reported indices. Regression analyses controlling for sex, age, family income, maternal education and medications were conducted.

**Results:**

demonstrated that youth and parents from all CP groups reported more sleep difficulties than youth without histories of CP. Participants from the CP, depressive symptoms and attention-hyperactivity problem group reported more sleep difficulty than all other groups, while their parents did not.

**Conclusions:**

These findings suggest that histories of CP, regardless of comorbidity, predispose youth to future sleep difficulties and highlight the importance of incorporating self and parental indices of sleep as well as examining the effect of comorbidity.

